# Supplementary material for: Growth promotion and antibiotic induced metabolic shifts in the chicken gut microbiome
Source: Commun Biol. 2022 Apr 1;5:293. doi: 10.1038/s42003-022-03239-6 (PMC8975857; doi:10.1038/s42003-022-03239-6)
Supplement: Supplementary file 2 — Supplementary Information [file 42003_2022_3239_MOESM2_ESM.pdf]

Supplementary Information

Growth promotion and antibiotic induced metabolic shifts in the chicken gut microbiome.

Germán Plata[a*], Nielson T. Baxter[a], Dwi Susanti[a], Alyssa Volland-Munson[a], Dharanesh Gangaiah[a], Akshitha Nagireddy[b], Shrinivasrao P. Mane[a], Jayanth Balakuntla[b], Troy B. Hawkins[a], Arvind Kumar (Mahajan)[a]

[a] Discovery research, Elanco Animal Health, Greenfield, Indiana 46140, USA

[b] Discovery research, Elanco Animal Health, Bangalore, 560008, India

[*] Correspondence to GP at german.plata_caviedes@elancoah.com

Supplementary Note 1

Our analysis of the virginiamycin cecum microbiome showed that the treatment increased the likelihood of observing high levels of *Salmonella* and *Escherichia* carrying multiple virulence and AMR genes. Although we saw a higher diversity and abundance of AMR genes after virginiamycin treatment, overall changes in AMR gene abundance were mild and in a similar direction as changes in overall gene content and taxonomic profiles. Thus, it is plausible that at the subtherapeutic dosages used in this study, the AMR profiles produced by different AGP reflect changes in microbial population structures, rather than specific selection for resistance. We note that AGP treatment sometimes resulted in lower AMR gene levels for certain AMR classes and that the types of AMR genes enriched by specific AGP did not necessarily match the AGP class. Johnson et al. [1] observed a negative correlation between bird weight and *Escherichia* loads in the trachea of 7-day old antibiotic-free birds. In our study, virginiamycin treated birds still showed increased performance compared to controls despite the much higher Enterobacteriaceae loads. So, even though inhibition of potential pathogens may contribute to AGP-mediated performance increases, it was probably not the main driver of this effect in our study.

Supplementary Note 2

In the process of characterizing the gene content of bacterial genera from the cecum, as expected, more genes were assigned through the metagenome annotations to the more abundant core genera (Spearman r: 0.49, p-value: 0.01). This is likely the effect of sequencing depth, as we found no significant correlation between the gene content of representative sequenced genomes from core genera and their mean abundance in the cecum (r: 0.12, p-value: 0.55). Given the incomplete gene coverage of core genera from metagenomics, we complemented the metagenome annotations using the metabolic gene content of fully sequenced bacterial genomes to obtain draft metabolic reconstructions of the expected size for each of the core genera. The resulting genus-level reconstructions ranged from 927 reactions for *Anaerotignum* to 1634 reactions for *Escherichia* (Supplementary Data 5). We noticed a positive correlation (r=0.64, p-value=6×10^{-4}) between the number of species with sequenced genomes in each core genera and the size of their corresponding reconstructions. This could possibly be a result of gut microbes with smaller genomes being less readily culturable and less often sequenced [2].

Supplementary Note 3

It was recently shown that mice fed antibiotics display lower levels of host supplied nitrogen in the large intestine, which was in part mediated by lower synthesis of mucus proteins by the intestinal epithelium [3]. Interestingly, following narasin treatment we observed a depletion of microbial genes involved in the metabolism of N-acetylneuraminate and hyaluronic acid, which are carbohydrate components of the intestinal epithelium extracellular matrix. Thus, it is possible that the cecum microbiome of narasin treated birds is less reliant on host derived carbohydrates and proteins. The concentration of both amino acids and dipeptides was lower in the cecum of narasin treated birds, which can be explained in multiple ways: higher absorption of amino acids and dipeptides by the host, lower degradation of host proteins by the cecum microbiome, or a lower production or higher utilization of these molecules by cecum microbes. However, the positive correlation trend observed between bird weights and amino acid and dipeptide levels in the serum and the negative correlation observed in the cecum of control birds suggest that nitrogen flux from the cecum to the host may positively impact performance. In the case of narasin treated birds, amino acid levels in the serum and amino acid and dipeptide levels in the cecum correlated positively with performance. Given the predicted ability of urea and ammonia utilization as main nitrogen sources, and assuming that the lower levels of amino acids and dipeptides in the cecum are due to reduced degradation of host proteins, the positive correlation observed in the cecum may reflect a higher supply by the microbiome of usable nitrogen in high performing birds.

Supplementary Note 4

In chickens, there is a limited capacity of the liver to excrete bilirubin, instead biliverdin produced by the catabolism of heme is the main bile pigment in bile [4]. Thus, it has been proposed that bilirubin is a product of bacterial reduction and dehydrogenation of biliverdin [5,6]. The observation of enriched tetrapyrrole metabolism, heme, siroheme, and cytochrome biosynthesis genes with narasin, further suggests that this AGP might have contributed to the synthesis and subsequent degradation of heme and related molecules directly in the cecum. Interestingly, heme degradation produces equimolar amounts of biliverdin and carbon monoxide (CO) [7] and the Wood-Ljungdahl Pathway, which was enriched by both BMD and narasin, consumes CO and CO_2 for acetogenesis, a potential source of metabolizable energy [8].

Supplementary Methods

Acquisition of metabolomics data

Samples were prepared using the automated MicroLab STAR® system from Hamilton Company. Samples were extracted with methanol under vigorous shaking for 2 min (Glen Mills GenoGrinder 2000) to precipitate protein and dissociate small molecules bound to protein or trapped in the precipitated protein matrix, followed by centrifugation to recover chemically diverse metabolites. The resulting extract was divided into four fractions: two for analysis by two separate reverse phase (RP) / Ultrahigh Performance Liquid Chromatography-Tandem Mass Spectroscopy (UPLC-MS/MS) methods using positive ion mode electrospray ionization (ESI), one for analysis by RP/UPLC-MS/MS using negative ion mode ESI, and one for analysis by HILIC/UPLC-MS/MS using negative ion mode ESI. Samples were placed briefly on a TurboVap® (Zymark) to remove the organic solvent. The sample extracts were stored overnight under nitrogen before preparation for analysis.

The sample extract was dried and reconstituted in solvents compatible to each of the four methods. One aliquot was analyzed using acidic positive ion conditions, chromatographically optimized for more hydrophilic compounds. In this method, the extract is gradient-eluted from a C18 column (Waters UPLC BEH C18-2.1x100 mm, 1.7 µm) using water and methanol, containing 0.05% perfluoropentanoic acid (PFPA) and 0.1% formic acid (FA). A second aliquot was also analyzed using acidic positive ion conditions but was chromatographically optimized for more hydrophobic compounds. In this method, the extract is gradient eluted from the C18 column using methanol, acetonitrile, water, 0.05% PFPA and 0.01% FA, and is operated at an overall higher organic content. A third aliquot was analyzed using basic negative ion optimized conditions using a separate dedicated C18 column. The basic extracts were gradient-eluted from the column using methanol and water, and with 6.5mM Ammonium Bicarbonate at pH 8. The fourth aliquot was analyzed via negative ionization following elution from a HILIC column (Waters UPLC BEH Amide 2.1x150 mm, 1.7 µm) using a gradient consisting of water and acetonitrile with 10mM Ammonium Formate, pH 10.8. The MS analysis alternates between MS and data-dependent MS^n scans using dynamic exclusion. The scan range varies slightly between methods, but covers approximately 70-1000 m/z.

UPLC-MS/MS was done using a Waters ACQUITY ultra-performance liquid chromatography (UPLC) and a Thermo Scientific Q-Exactive high resolution/accurate mass spectrometer interfaced with a heated electrospray ionization (HESI-II) source and Orbitrap mass analyzer operated at 35,000 mass resolution.

Raw data was extracted, peak-identified, and QC processed using Metabolon's hardware and software. Compounds were identified by comparison to library entries of purified standards. The library, based on authenticated standards, contains the retention time/index (RI), mass to charge ratio (*m/z)*, and chromatographic data (including MS/MS spectral data) on all molecules present in the library. Biochemical identifications were based on three criteria: retention index within a narrow RI window of the proposed identification, accurate mass match to the library +/- 10 ppm, and the MS/MS forward and reverse scores. MS/MS scores are based on a comparison of the ions present in the experimental spectrum to ions present in the library entry spectrum.

Supplementary Tables

Supplementary Table 1. Diet formulations used in the study.

Ingredients (%)	Starter (0 – 14 D)	Grower (14 – 28 D)	Finisher (28 – 35 D)
Corn	55.92	57.46	60
Soybean meal	37.97	35.42	32.73
Soybean oil	2.11	3.72	4.43
L-Lysine HCL	0.31	0.17	0.03
DL-Methionine	0.31	0.25	0.21
L-Threonine	0.15	0.1	0.04
Dicalcium phosphate 18.5%	1.28	1.03	0.82
Calcium carbonate	1.23	1.12	1.01
Salt	0.35	0.35	0.35
Promote phytase 2500	0.03	0.03	0.03
Provimi 5 PMX	0.3	0.3	0.3
MycoCURB	0.05	0.05	0.05
**Calculated Analyses**			
Metabolizable Energy (kcal/kg)	3,018	3,134	3,204
Crude Protein (%)	22	20.7	19.4
Digestible Lys (%)	1.37	1.19	1.02
Digestible Met (%)	0.64	0.58	0.52
Digestible Thr (%)	0.86	0.77	0.68
Ca (%)	0.95	0.85	0.76
Available P (%)	0.48	0.43	0.39

Supplementary Table 2. Manufacturers, dose ranges and target doses of the four AGP used in the study.

	BMD	Avilamycin	Virginiamycin	Narasin
Product name	BMD-50	Intepríty	Stafac-20	Monteban-45
Manufacturer	Zoetis	Elanco	Phibro	Elanco
Abx. concentration (g. abx. / lb. product)	50	45.4	20	45
Recommended dose range (g. abx. / ton feed)	4 to 50	13.6 to 40.9	5 to 20	54 to 90
Target dose (g. abx. / ton feed)	50	20	16.5	70
Feed preparation (lb. product / ton feed)	1	0.44	0.83	1.56

Supplementary Table 3. Universal single copy genes with 1 or fewer estimated orthologous gene displacements as determined by Creevey et al.[9]

COG	KO	Role
COG0048	K02950	SSU ribosomal protein S12p (S23e)
COG0052	K02967	SSU ribosomal protein S2p (SAe)
COG0080	K02867	LSU ribosomal protein L11p (L12e)
COG0085	K03043	DNA-directed RNA polymerase beta subunit (EC 2.7.7.6)
COG0087	K02906	LSU ribosomal protein L3p (L3e)
COG0091	K02890	LSU ribosomal protein L22p (L17e)
COG0092	K02982	SSU ribosomal protein S3p (S3e)
COG0093	K02874	LSU ribosomal protein L14p (L23e)
COG0094	K02931	LSU ribosomal protein L5p (L11e)
COG0096	K02994	SSU ribosomal protein S8p (S15Ae)
COG0097	K02933	LSU ribosomal protein L6p (L9e)
COG0100	K02948	SSU ribosomal protein S11p (S14e)
COG0184	K02956	SSU ribosomal protein S15p (S13e)
COG0200	K02876	LSU ribosomal protein L15p (L27Ae)
COG0201	K03076	Protein translocase subunit SecY
COG0052	K03110	Signal recognition particle receptor FtsY
COG0088	K02926	LSU ribosomal protein L4p (L1e)
COG0098	K02988	SSU ribosomal protein S5p (S2e)
COG0099	K02952	SSU ribosomal protein S13p (S18e)
COG0103	K02996	SSU ribosomal protein S9p (S16e)
COG0185	K02965	SSU ribosomal protein S19p (S15e)
COG0186	K02961	SSU ribosomal protein S17p (S11e)
COG0197	K02878	LSU ribosomal protein L16p (L10e)
COG0522	K02986	SSU ribosomal protein S4p (S9e)

Supplementary Figures

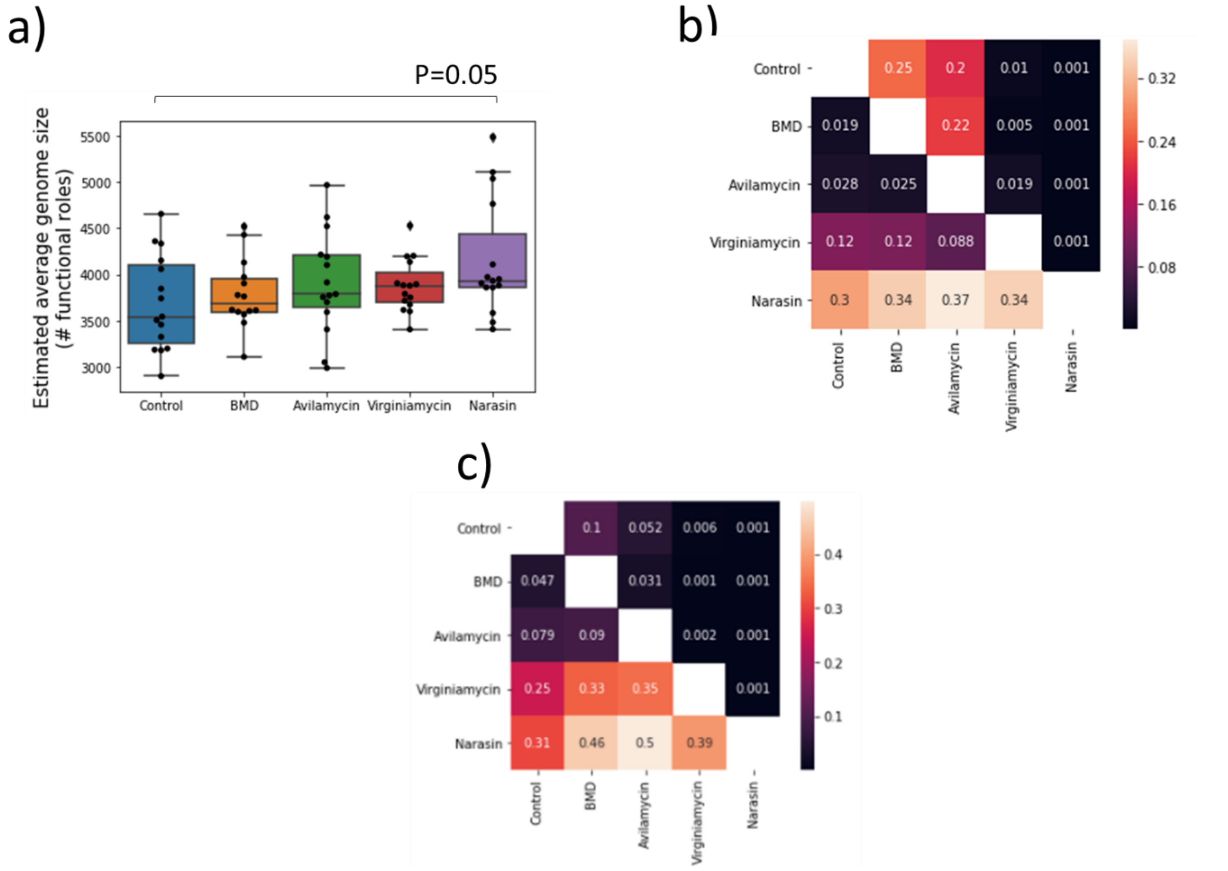

Supplementary Figure 1. Functional and taxonomic microbiome differences from shotgun metagenomics data. a) Estimated mean genome sizes per treatment group. Mean genome sizes were estimated as the sum of the abundances of genes assigned different functional roles, normalized by the abundance of universal single copy genes. The boxes represent the median and interquartile range; whiskers indicate the range of the distribution excluding outliers. Outliers are at least 1.5 times the interquartile range below or above the first and third quartile, respectively. The p-value is for the two-sided Mann-Whitney U test. **b)** ANOSIM results for the comparison of functional profiles across treatment groups. Values above the diagonal represent ANOSIM p-values. Values below the diagonal represent ANOSIM R-scores. **c)** Like b, but for the comparison of species-level taxonomic profiles obtained from the shotgun metagenomics data.

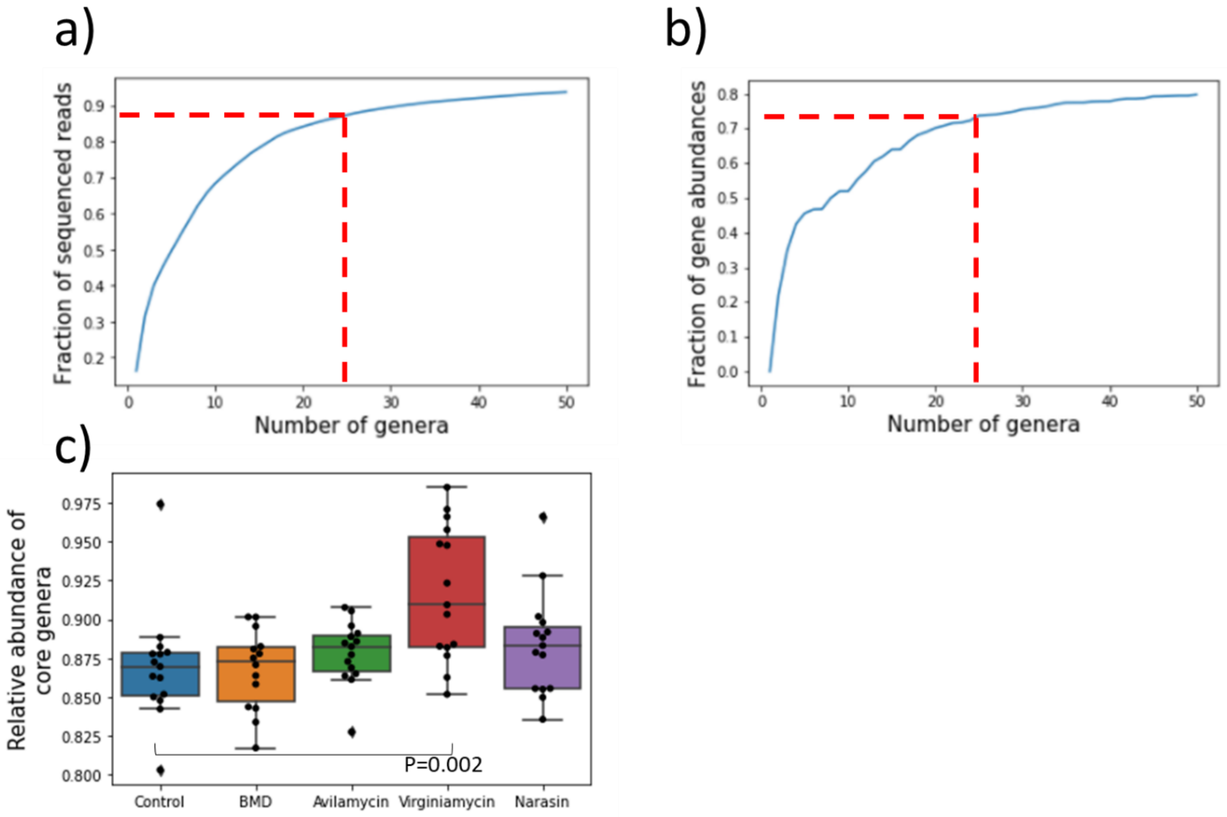

Supplementary Figure 2. Core genera of the broiler cecum microbiome. Bacterial genera identified from metagenomics reads were sorted according to their average relative abundance across samples. The figures show the mean fraction of classified reads (**a**) or functional role relative abundances (**b**) accounted for the top n most abundant genera. The mean is calculated across all 74 samples from day 35 broilers. The dashed red lines show that the top 25 most abundant genera account for ~90% of classified reads and ~75% of gene abundances. **c)** Relative abundance of core genera as a function of treatment. The p-value is for the two-sided Mann-Whitney U test. Boxes represent the median and interquartile range; whiskers indicate the range of the distribution excluding outliers.

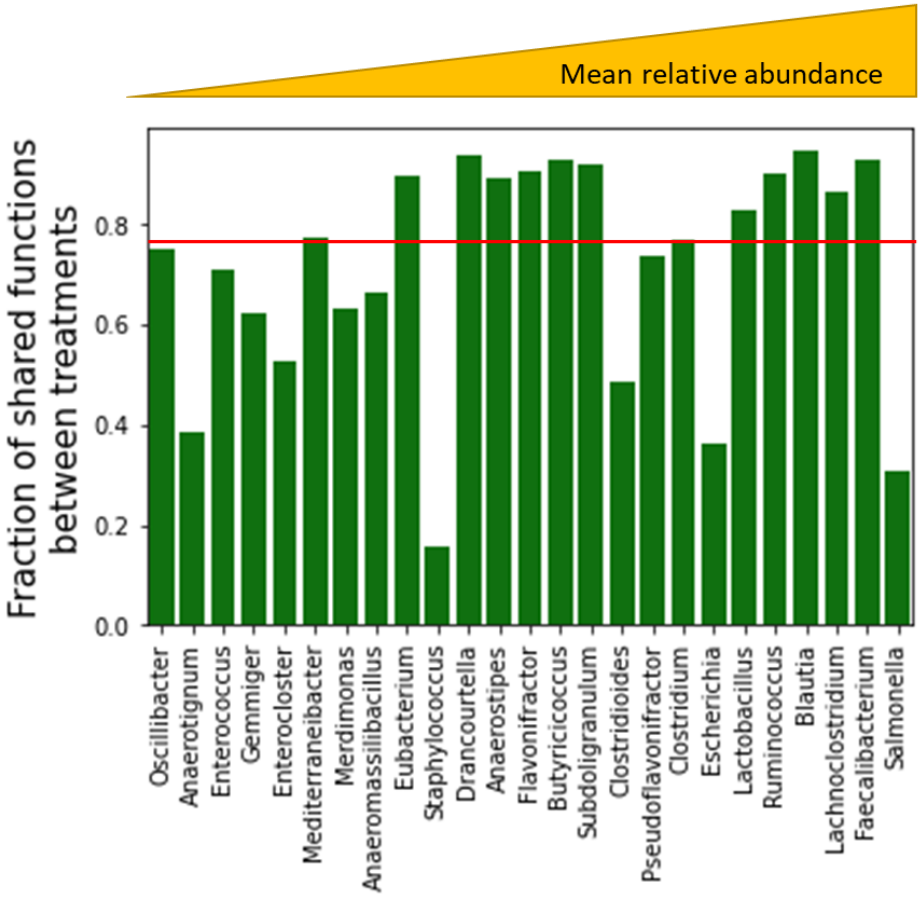

Supplementary Figure 3. Functional roles assigned to core genera shared between samples from different treatments. The figure shows the median overlap between assigned functional roles to each treatment for all pairwise treatment comparisons. Data are shown for each of 25 core genera sorted by their mean abundance across all samples. Overlap was calculated as the number of shared roles between treatments divided by the number of roles in the treatment with the lowest relative abundance for the corresponding genus. The red line indicates the median overlap (0.77).

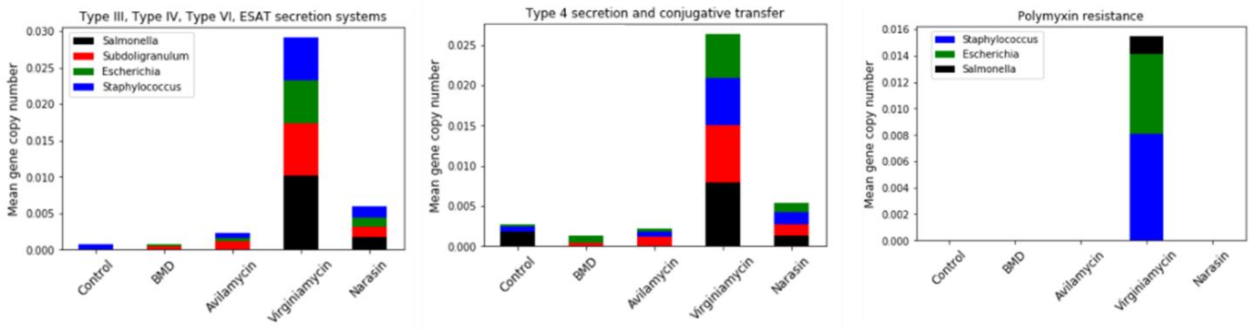

Supplementary Figure 4. Contribution to virulence and AMR terms enriched by virginiamycin by core genera. Each plot corresponds to a virginiamycin enriched functional term. The y-axis shows the mean abundance associated with core genera (in units of mean copy numbers per genome) of genes in the corresponding function. Results for each treatment represent the mean across samples in that treatment. Colors indicate the contribution to function abundances by genes assigned to the corresponding core genera.

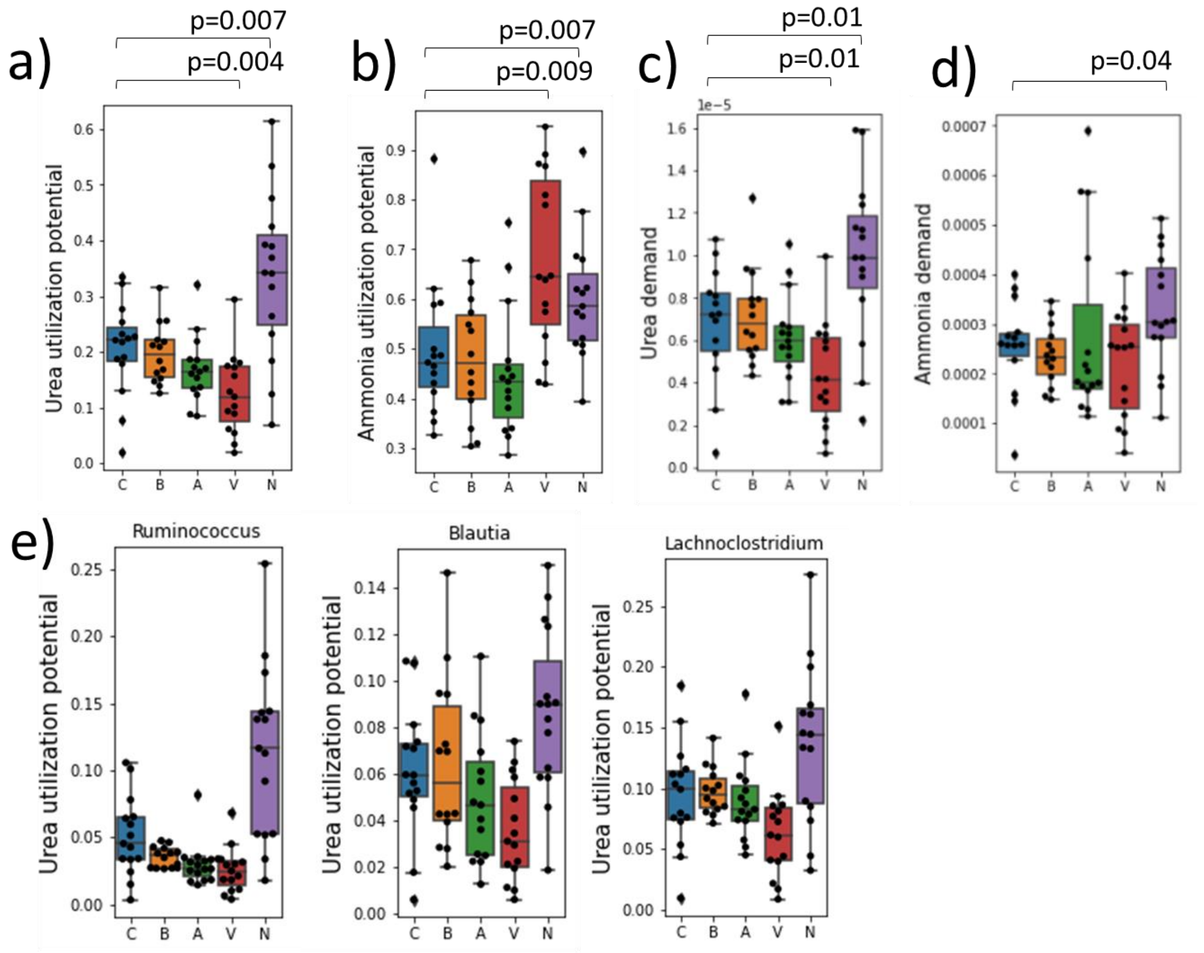

Supplementary Figure 5. Urea and ammonia utilization potential and essentiality for the core cecum microbiome across treatments. a) The potential for urea utilization as a main nitrogen source for biomass synthesis by the core cecum microbiome. **b)** Like a, but for the ability of using ammonia as a main nitrogen source. **c)** The metabolic demand of urea as a nitrogen source for growth. Values represent the probability that urea is essential for growth for a random member of the core cecal microbiome. **d)** like c but for the essentiality of ammonia as a nitrogen source. **e)** The contribution of individual genera from the core cecum microbiome to the utilization potential of urea as a main nitrogen source. Values represent the relative abundance in the core of genera predicted to use urea as a nitrogen source for growth. The p-values are for the two-sided Mann-Whitney U test. Boxes represent the median and interquartile range; whiskers indicate the range of the distribution excluding outliers. C: control. B: BMD, A: avilamycin, V: virginiamycin, N: narasin.

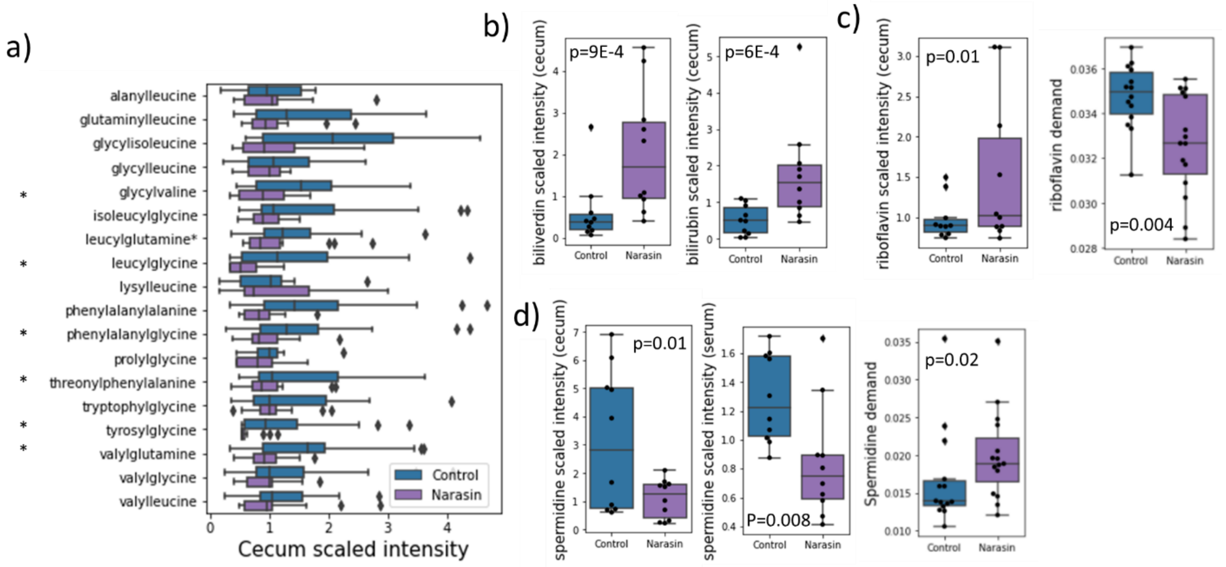

Supplementary Figure 6. Differentially abundant metabolites in the cecum of narasin treated birds. **a)** Scaled intensities of dipeptides in the cecum of control and narasin treated birds. *p-value<0.05. **b)** Scaled intensities for the concentration of biliverdin and bilirubin in the cecum. **c)** Scaled intensities for the concentration of riboflavin in the cecum and estimated riboflavin demand by the core cecum microbiome in control and narasin treated birds. **d)** Scaled intensities for the concentration of spermidine in the cecum and serum and estimated spermidine demand by the core cecum microbiome in control and narasin treated birds. The p-values for metabolite scaled intensities are for Welch's T test, p-values for estimated metabolite demands are for the Mann-Whitney U test. Boxes represent the median and interquartile range; whiskers indicate the range of the distribution excluding outliers.

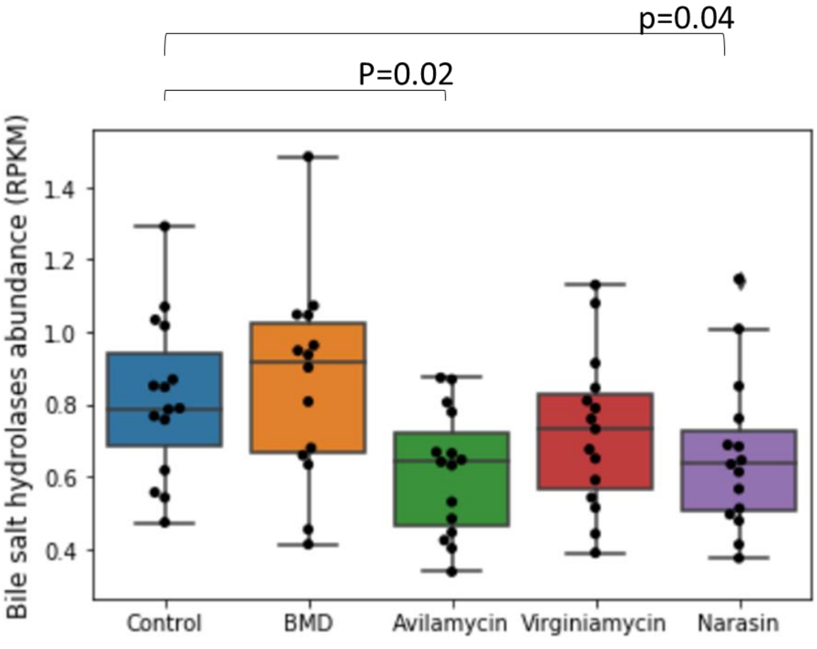

Supplementary Figure 7. Abundance of bile salt hydrolase homologues across treatments. Values are expressed as reads per kilobase per million reads (RPKM) corresponding to reads mapped to regions in assembled contigs with homology to bile salt hydrolases. BLASTX [10] was used to identify regions in the assembled contigs for each sample with >50% sequence identity over 70% of the length of UniProt [11] proteins annotated as bile salt hydrolases or chloroylglycine hydrolases. The indicated p-value is for the two-sided Mann-Whitney U test. Boxes represent the median and interquartile range; whiskers indicate the range of the distribution excluding outliers.

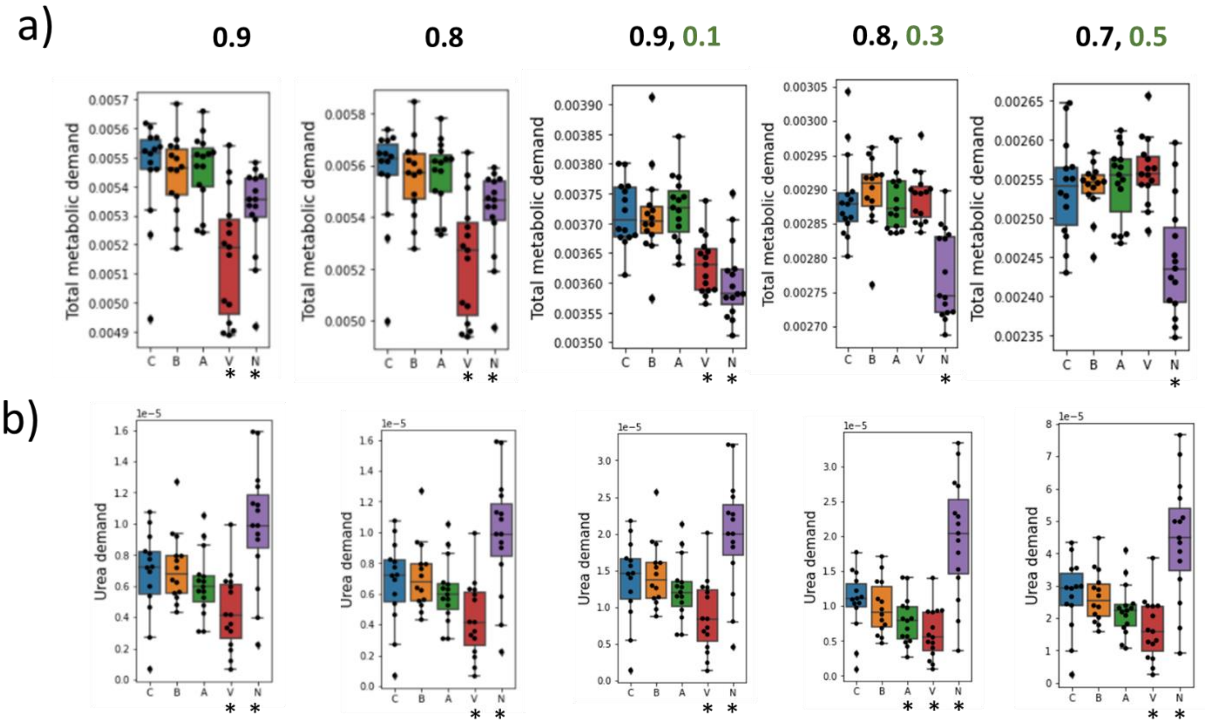

Supplementary Figure 8. Comparison of metabolic demand predictions for different parameters in the simulation of *in silico* media. Results are shown for the average metabolite essentiality for growth of the core microbiome (**a**), and for the average essentiality of urea for microbial growth (**b**). The columns show results for different parameter combinations used for simulating random *in silico* media to test essentiality. Data are shown for 90% and 80% probabilities of metabolites with transporters being included in the *in silico* media (columns 1 and 2), as well as 90, 80% and 70% probabilities for metabolites with transporters combined with 10%, 30% or 50% probabilities for metabolites without transporters (columns 3, 4 and 5). Boxes represent the median and interquartile range; whiskers indicate the range of the distribution excluding outliers. C: control, B: BMD, A: avilamycin, V: virginiamycin, N: narasin.

Supplementary References

1 Johnson, T. J. *et al.* A Consistent and Predictable Commercial Broiler Chicken Bacterial Microbiota in Antibiotic-Free Production Displays Strong Correlations with Performance. *Appl Environ Microbiol* **84**, doi:10.1128/AEM.00362-18 (2018).

2 Garza, D. R. & Dutilh, B. E. From cultured to uncultured genome sequences: metagenomics and modeling microbial ecosystems. *Cell Mol Life Sci* **72**, 4287-4308, doi:10.1007/s00018-015-2004-1 (2015).

3 Reese, A. T. *et al.* Microbial nitrogen limitation in the mammalian large intestine. *Nat Microbiol* **3**, 1441-1450, doi:10.1038/s41564-018-0267-7 (2018).

4 Lin, G. L., Himes, J. A. & Cornelius, C. E. Bilirubin and biliverdin excretion by the chicken. *Am J Physiol* **226**, 881-885, doi:10.1152/ajplegacy.1974.226.4.881 (1974).

5 Zaefarian, F., Abdollahi, M. R., Cowieson, A. & Ravindran, V. Avian Liver: The Forgotten Organ. *Animals (Basel)* **9**, doi:10.3390/ani9020063 (2019).

6 Bell, D. J. & Freeman, B. M. *Physiology and biochemistry of the domestic fowl. Volumes 1, 2, 3*. (London, UK, Academic Press, Inc., 1971).

7 Landaw, S. A., Callahan, E. W., Jr. & Schmid, R. Catabolism of heme in vivo: comparison of the simultaneous production of bilirubin and carbon monoxide. *J Clin Invest* **49**, 914-925, doi:10.1172/JCI106311 (1970).

8 Fievez, V., Mbanzamihigo, L., Piattoni, F. & Demeyer, D. Evidence for reductive acetogenesis and its nutritional significance in ostrich hindgut as estimated from in vitro incubations. *J Anim Physiol Anim Nutr (Berl)* **85**, 271-280, doi:10.1046/j.1439-0396.2001.00320.x (2001).

9 Creevey, C. J., Doerks, T., Fitzpatrick, D. A., Raes, J. & Bork, P. Universally distributed single-copy genes indicate a constant rate of horizontal transfer. *PLoS One* **6**, e22099, doi:10.1371/journal.pone.0022099 (2011).

10 Altschul, S. F. *et al.* Gapped BLAST and PSI-BLAST: a new generation of protein database search programs. *Nucleic Acids Res* **25**, 3389-3402, doi:10.1093/nar/25.17.3389 (1997).

11 UniProt, C. UniProt: the universal protein knowledgebase in 2021. *Nucleic Acids Res* **49**, D480-D489, doi:10.1093/nar/gkaa1100 (2021).
