# Supplementary material for: Growth promotion and antibiotic induced metabolic shifts in the chicken gut microbiome
Source: Commun Biol. 2022 Apr 1;5:293. doi: 10.1038/s42003-022-03239-6 (PMC8975857; doi:10.1038/s42003-022-03239-6)
Supplement: Supplementary file 3 — Description of Additional Supplementary Files [file 42003_2022_3239_MOESM3_ESM.pdf]

Description of Additional Supplementary Files

Supplementary Data 1.

Comparison of weekly average daily gain and feed conversion ratios between treatments

Supplementary Data 2.

Genus-level differential relative abundances in the cecum of day 35 birds between AGP treatments and control Page 6 of 16

Supplementary Data 3.

GSEA results for the differential mean copy number of genes in the cecum of AGP treated birds compared to control

Supplementary Data 4.

GSEA results for the correlation between mean gene copy numbers in the cecum and the day 35 weight of birds in each treatment group

Supplementary Data 5.

Core genera annotation and metabolic reconstruction statistics

Supplementary Data 6.

Differentially abundant metabolites in the cecum and serum of narasin treated birds

Supplementary Data 7.

GSEA results for differentially abundant metabolites in AGP treated birds compared to control

Supplementary Data 8.

GSEA results for the correlation between metabolite abundances and day 35 weight in control and narasin treated birds

Supplementary Data 9.

Raw metabolomics data for cecal and serum samples in the control and narasin treatments Page 7 of 16

Supplementary Data 10.

Pathway classification of cecum and serum metabolites used for GSEA

Supplementary Data 11.

Source data formain manuscript figures
