# Supplementary material for: Growth promotion and antibiotic induced metabolic shifts in the chicken gut microbiome
Source: Commun Biol. 2022 Apr 1;5:293. doi: 10.1038/s42003-022-03239-6 (PMC8975857; doi:10.1038/s42003-022-03239-6)
Supplement: Supplementary file 15 — Reporting Summary [file 42003_2022_3239_MOESM15_ESM.pdf]

nature portfolio

Corresponding author(s): Germán Plata
Last updated by author(s): Feb 14, 2022

Reporting Summary

Nature Portfolio wishes to improve the reproducibility of the work that we publish. This form provides structure for consistency and transparency in reporting. For further information on Nature Portfolio policies, see our Editorial Policies and the Editorial Policy Checklist.

Statistics

For all statistical analyses, confirm that the following items are present in the figure legend, table legend, main text, or Methods section.

n/a	Confirmed	
☐	☒	The exact sample size (*n*) for each experimental group/condition, given as a discrete number and unit of measurement
☐	☒	A statement on whether measurements were taken from distinct samples or whether the same sample was measured repeatedly
☐	☒	The statistical test(s) used AND whether they are one- or two-sided<br>*Only common tests should be described solely by name; describe more complex techniques in the Methods section.*
☐	☒	A description of all covariates tested
☐	☒	A description of any assumptions or corrections, such as tests of normality and adjustment for multiple comparisons
☐	☒	A full description of the statistical parameters including central tendency (e.g. means) or other basic estimates (e.g. regression coefficient) AND variation (e.g. standard deviation) or associated estimates of uncertainty (e.g. confidence intervals)
☒	☐	For null hypothesis testing, the test statistic (e.g. *F*, *t*, *r*) with confidence intervals, effect sizes, degrees of freedom and *P* value noted<br>*Give P values as exact values whenever suitable.*
☒	☐	For Bayesian analysis, information on the choice of priors and Markov chain Monte Carlo settings
☒	☐	For hierarchical and complex designs, identification of the appropriate level for tests and full reporting of outcomes
☐	☒	Estimates of effect sizes (e.g. Cohen's *d*, Pearson's *r*), indicating how they were calculated

Our web collection on statistics for biologists contains articles on many of the points above.

Software and code

Policy information about availability of computer code

Data collection	*Provide a description of all commercial, open source and custom code used to collect the data in this study, specifying the version used OR state that no software was used.*
Data analysis	cutadapt (v. 2.5), Trimmomatic (v 0.39), DADA2 (v.1.12.1), Kaiju (v. 1.7.3), metametamerge (v. 1.1), Spades (v. 3.13.1), Prokka (v. 1.14.5), Diamond (v. 0.9.30), BWA (v. 0.7.17), HTseq (v. 0.11.3), and AMR++ (v. 2.0.2) were used to process and annotate sequenced reads. scikit-bio (v. 0.5.6), sicpy (v. 1.3.1), scikit-learn (v. 0.21.3), gseapy (v. 0.10.2) metamicrobiomeR(v1.2) and the cobrapy toolbox (v. 0.19.0) were used to analyze the data. Custom code used for flux analysis is available at https://github.com/platyias/BroilerCoreModels or doi:10.5281/zenodo.6083554.

For manuscripts utilizing custom algorithms or software that are central to the research but not yet described in published literature, software must be made available to editors and reviewers. We strongly encourage code deposition in a community repository (e.g. GitHub). See the Nature Portfolio guidelines for submitting code & software for further information.

Data

Policy information about availability of data

All manuscripts must include a data availability statement. This statement should provide the following information, where applicable:

- Accession codes, unique identifiers, or web links for publicly available datasets
- A description of any restrictions on data availability
- For clinical datasets or third party data, please ensure that the statement adheres to our policy

Raw 16S and shotgun metagenomics sequences were deposited in the SRA database under accession number PRJNA751585. Draft metabolic reconstructions for the 25 core genera and processed taxonomic and functional profiles are available from (https://github.com/platyias/BroilerCoreModels).

Field-specific reporting

Please select the one below that is the best fit for your research. If you are not sure, read the appropriate sections before making your selection.

☐ Life sciences ☐ Behavioural & social sciences ☒ Ecological, evolutionary & environmental sciences

For a reference copy of the document with all sections, see nature.com/documents/nr-reporting-summary-flat.pdf

Ecological, evolutionary & environmental sciences study design

All studies must disclose on these points even when the disclosure is negative.

Study description	Broilers from a commercial hatchery were randomly assigned to one of 25 floor pens (20 animals per pen). Groups of 5 pens were assigned to each of 5 different treatments.
Research sample	500 healthy Ross 708 male broilers. The number of birds per pen was selected to approximate bird density under commercial rearing conditions.
Sampling strategy	On days 7, 21 and 35 of the study three birds per pen were randomly selected and euthanized for the collection of cecal contents and blood.
Data collection	Individual body weights , pen weights and feed weigh backs were collected by a contract research organization in Indiana. Necropsy samples were collected and frozen for sequencing of metabolomics processing.
Timing and spatial scale	Individual body weights were collected on days 0, 7, 21 and 35. Pen weights were collected on days 14 and 28. Feed weigh backs for each pen were done on days 7, 14, 21, 28 and 35 of the study. 16S amplicon sequencing data were obtained for day 7, 21 and 35 samples. Shotgun metagenomics and metabolomics data were obtained for day 35 samples.
Data exclusions	Samples with less than 10,000 sequencing reads were not considered for analysis.
Reproducibility	Conclusions are based upon multiple lines of analysis, namely, taxonomic profiles from both 16S and shotgun metagenomics, gene abundance quantification and metabolic modeling results, as well as untargeted metabolomics measurements.
Randomization	Allocation of individual tags to birds and of pens to treatments was done at random. The 3 birds per pen used for microbiome sampling were chosen sequentially based on tag ids.
Blinding	Treatment group data was not considered during data collection.

Did the study involve field work? ☐ Yes ☒ No

Reporting for specific materials, systems and methods

We require information from authors about some types of materials, experimental systems and methods used in many studies. Here, indicate whether each material, system or method listed is relevant to your study. If you are not sure if a list item applies to your research, read the appropriate section before selecting a response.

Materials & experimental systems

n/a	Involved in the study	
☒	☐	Antibodies
☒	☐	Eukaryotic cell lines
☒	☐	Palaeontology and archaeology
☐	☒	Animals and other organisms
☒	☐	Human research participants
☒	☐	Clinical data
☒	☐	Dual use research of concern

Methods

n/a	Involved in the study	
☒	☐	ChIP-seq
☒	☐	Flow cytometry
☒	☐	MRI-based neuroimaging

Animals and other organisms

Policy information about studies involving animals; ARRIVE guidelines recommended for reporting animal research

Laboratory animals	Ross 708 male broilers with an approximate weight of 35 to 45 g at the start of the study that received a 1x dose of Coccivac®-B52 vaccine (Merck Animal Health)
Wild animals	*Provide details on animals observed in or captured in the field; report species, sex and age where possible. Describe how animals were caught and transported and what happened to captive animals after the study (if killed, explain why and describe method; if released,*

say where and when) OR state that the study did not involve wild animals.

Field-collected samples	*For laboratory work with field-collected samples, describe all relevant parameters such as housing, maintenance, temperature, photoperiod and end-of-experiment protocol OR state that the study did not involve samples collected from the field.*
Ethics oversight	The live animal experiment and procedures were approved by Elanco institutional animal care committee, approval number IACUC # EIAC-0773.

Note that full information on the approval of the study protocol must also be provided in the manuscript.
